# Interplay between Dietary Polyphenols and Oral and Gut Microbiota in the Development of Colorectal Cancer

**DOI:** 10.3390/nu12030625

**Published:** 2020-02-27

**Authors:** Carolina Cueva, Mariana Silva, Iris Pinillos, Begoña Bartolomé, M. Victoria Moreno-Arribas

**Affiliations:** Institute of Food Science Research (CIAL), CSIC-UAM, C/Nicolás Cabrera 9, Campus de Cantoblanco, 28049 Madrid, Spain; carolina.cueva@csic.es (C.C.); mariana.silva@csic.es (M.S.); i.pinillos@csic.es (I.P.); b.bartolome@csic.es (B.B.)

**Keywords:** colorectal cancer, oral microbiota, gut microbiota, dysbiosis, diet, polyphenol metabolites

## Abstract

Colorectal cancer (CRC) is the third most diagnosed type of cancer worldwide. Dietary features play an important role in its development, and the involvement of human microbial communities in this pathology has also recently been recognized. Individuals with CRC display alterations in gut bacterial composition and a notably higher abundance of putative oral bacteria in colonic tumors. Many experimental studies and preclinical evidence propose that dietary polyphenols have a relevant role in CRC development and progression, mainly attributed to their immunomodulatory activities. Furthermore, polyphenols can modulate oral and gut microbiota, and in turn, intestinal microbes catabolize polyphenols to release metabolites that are often more active and better absorbed than the original phenolic compounds. The current study aimed to review and summarize current knowledge on the role of microbiota and the interactions between dietary polyphenols and microbiota in relation to CRC development. We have highlighted the mechanisms by which dietary polyphenols and/or their microbial metabolites exert their action on the pathogenesis and prevention of CRC as modulators of the composition and/or activity of oral and intestinal microbiota, including novel screening biomarkers and possible nutritional therapeutic implications.

## 1. Introduction

Cancer is defined as an uncontrolled proliferation of malignant cells in a host and is one of the main causes of death worldwide. According to the World Health Organization’s (WHO) projections, there will be more than 21 million new cases of cancer and more than 13 million cancer-related deaths worldwide by 2030 [1]. Concerning scientific strategies, it is foreseeable that ‘Cancer’ will be one of the ‘Mission areas’ of the Horizon Europe Programme for Research and Innovation 2021–2027 of the European Union, together with Climate Change, Climate-neutral and Smart Cities, Healthy Seas and Oceans, Coastal and Inland Waters, and Soil Health and Food. As such, efforts will be made in the next years in order to promote policies and transformative solutions in cancer prevention, prediction, detection, diagnosis, and treatment. 

Colorectal cancer (CRC) is the third most common cause of cancer mortality in the world and is one of the most commonly malignant tumors and forms of diagnosed cancer, accounting for about 1.2 million new cases and 600,000 deaths per year [1,2]. The CRC etiology is still not fully understood, but the increased incidence of CRC in the last decades can be explained by population aging, the modification of diets and eating habits, and genetic and epigenetic variations. The majority of cases are due to sporadic cancers (75%) that could be influenced by poor dietary patterns, host immunity, and lifestyle factors such as smoking, low physical activity, and obesity [3,4,5]. Other gastrointestinal disorders, such as inflammatory bowel disease (IBD), characterized by chronic inflammation, mucosa disruption, and the excessive production of reactive oxygen species (ROS), act as risk factors in cancer onset, progression, and metastatic diffusion [6]. In recent years, a new and remarkable factor in the development of cancer and other related intestinal diseases has emerged; the gastrointestinal tract microbiota [7,8].

The human body harbors trillions of microbial cells whose coordinated actions are believed to be important for human life. Such microbial cell populations reach their highest density in the intestinal compartment, where they collectively form a complex microbial community known as the gut microbiota that develops over the course of host infancy to eventually reach its adult form. The microbiota of the human intestinal tract harbors more than 100,000 billion microorganisms, including bacteria, fungi, viruses, protozoa, and archaea, with bacteria representing a majority [9,10]. This is an extremely complex ecosystem; in particular, the human adult gut microbiota is estimated to comprise over 1000 different bacterial species with more than 7000 strains [11,12]. According with the anatomy of the gastrointestinal tract, several environments characterized by specific microbiota compositions are found. The microbiome (collective genome of the microbiota) with a size larger than that of the human genome, is considered to act as a virtual organ that participates in the physiological functioning of the host [13]. Within the functions of intestinal microbes in human physiology, we find their contribution to the metabolism of nutrients and xenobiotics (for example, synthesis of vitamins, digestion of oligo and polysaccharides, drugs, etc.) and the regulation of immune and neuroendocrine functions [14]. Some of these effects are mediated by products of bacterial metabolism, such as short-chain fatty acids (SCFA), including acetate, butyrate, or propionate, which influence the gut barrier, the inflammatory tone, and the metabolic homeostatic control in different tissues [15].

As the gut microbiota has a well-established role in host homeostasis, several highly prevalent gastrointestinal diseases have been associated with imbalances in microbiota composition (termed dysbiosis) [16]. These human diseases include autoimmune and autoinflammatory disorders, such as allergies, obesity, inflammatory bowel disease [17], and more recently, the association with cancer cases has been suggested [18]. Microbes have been implicated in the pathogenesis of several human cancers, most strikingly in the case of *Helicobacter pylori* and gastric carcinoma and some gastric lymphomas [19]. *H. pylori* is now designated a gastric carcinogen and a preclinical risk factor. In relation to CRC, two complex environments of the gastrointestinal tract have been the most studied: The oral cavity and intestinal region. In the first case, oral microbial alterations linked to CRC have been suggested, such as higher abundance of putative oral bacteria on colonic tumors [20]. In addition, disturbances in the expression of the genes of the oral mucosa cells have been proposed as factors contributing to the development of cancer; meanwhile, in the case of the intestine, a reduction in the production of protective mucin and peptide antimicrobials, facilitating the exposure to mutagenic metabolites and colonization by pathogens, has been proposed [21]. However, it is still unclear whether the general features of a healthy/dysbiosis microbiota can be defined at population level; CRC seems to be accompanied by shifts in an individual’s normal microbiota toward a dysbiotic composition which could aggravate disease pathogenesis, creating a vicious circle, though this is not completely proven [22]. 

As previously mentioned, CRC is recognized as a multifactorial disease that depends on environmental variables as well as intrinsic individual factors. In particular, dietary habits have been linked to alterations in the intestinal microbiota that could also contribute to the pathophysiology underlying CRC and its metabolic and psychological complications. Among bioactive compounds of diet, polyphenols are known to exert favorable effects on free radicals, inflammation, and gut microbiota (composition and functionality modulation) [23,24]. These compounds could be good candidates for cancer prevention and treatment, targeting key molecular pathways involved in CRC [25,26]. For this reason, it is important to identify the potential interplaying factors surrounding the interrelationships of polyphenols–microbiota and CRC, and as such, pose a specific target to the development of potential nutritional and pharmaceutical approaches. 

In this review, we focus on current knowledge on the role of microbiota and the interactions between dietary polyphenols and microbiota in relation to CRC development. With this purpose, a systematic literature review from three electronic databases: Web of Science, PubMed, and Scopus, for studies of the last 10 years, has been carried out. Searches were conducted using the terms “colorectal cancer” and “polyphenol” and “microbiota” or “gut” or “oral” or “metabolism”. Further relevant studies were identified through manual searches of reference lists of selected studies and recently published review articles. In the last years, there has been an increase in publications related to these issues, most of them propose the modulation of the microbiota as a new therapeutic target against CRC. Within this framework, and taking into account that the main microenvironments of the gastrointestinal tract related to CRC are the oral and intestinal microbiota, we emphasize the mechanisms by which dietary polyphenols and/or their microbial metabolites exert their action on the pathogenesis and prevention of CRC as modulators of the composition and/or activity of oral and intestinal microbiota.

## 2. Oral and Intestinal Microbiota and Colorectal Cancer

Due to its frequent communication with the external environment, the oral cavity is perhaps one of the most complex ecosystems in the body. So far, 770 taxa, mostly belonging to the bacteria taxonomic group, have been identified. The major phyla of oral bacteria comprise *Firmicutes* (genus *Gemella*, *Granulicatella*, *Streptococcus* and *Veillonella*), *Bacteroidetes* (strongly represented by *Prevotella*), *Proteobacteria* (genus *Neisseria, Haemophilus*), *Actinobacteria* (genus *Corynebacterium*, *Rothia*, *Actinomyces*), and *Fusobacteria* (genus *Fusobacterium*) [27,28]. The roles of these microorganisms include digestion of food, resisting pathogens, maintaining homeostasis, and modulating the immune system, contributing in this way oral and general well-being. Despite this, they are also responsible for a variety of oral diseases [29,30]. 

In the following intestine anatomical regions, the gut microbiota changes according to physiology, pH and O_2_ tension, digest flow rates, substrate availability, and host secretions [31]. Under healthy conditions, the predominant bacteria in the acidic gastric environment (median pH 1.4) belong to the phyla of *Actinobacteria*, *Bacteroidetes*, *Firmicutes* (the predominant genus is *Streptococcus*), and *Proteobacteria* (which include *Helicobacter pylori*) [32]. With regard to the small intestine, it provides a more challenging environment for microbial colonizers given the fairly short transit times (3–5 h) and the high bile concentrations; meanwhile, the large intestine, which is characterized by slow flow rates and neutral to mildly acidic pH, harbors by far the largest microbial community (dominated by obligate anaerobic bacteria). The dominant gut bacterial phyla are the *Firmicutes* (including *Clostridium*, *Enterococcus*, *Lactobacillus*, and *Ruminococcus* genera) and *Bacteroidetes* (including *Bacteroides* and *Prevotella* genera). Other subdominant or minor phyla include *Actinobacteria*, *Proteobacteria*, *Fusobacteria*, and *Verrucomicrobia* [11]. The main functions performed by gut microbiota are trophic (i.e., the control of epithelial cell proliferation and differentiation), metabolic (i.e., the fermentation of indigestible food components into absorbable metabolites), and protective (i.e., the out-competition of pathogens) that are essential to maintain human health status. As the gut microbiota has a well-established role in host homeostasis, several highly prevalent gastrointestinal diseases, including cancer, have been associated with dysbiosis in microbiota composition [33]. 

In the case of oral microbiota, recent scientific evidences suggest its association with oral cancer as well as other gastrointestinal cancers, such as esophageal cancer, colorectal cancer, and pancreatic cancer [28]. In particular, the esophageal and pancreatic cancers have been associated with an abundance of the periodontal pathogens *Streptococcus anginosus* and *Tannerella forsythia* and *Porphyromonas gingivalis* and *Aggregatibacter actinomycetemcomitans*, respectively [22,34]. However, for this review, our interest will be focused on one of the most common gastrointestinal cancers, CRC. Although evidence is very new, studies in this area explored the potential involvement of oral and intestinal microbiota in the different stages of this tumor [35,36]. 

**Figure 1:**
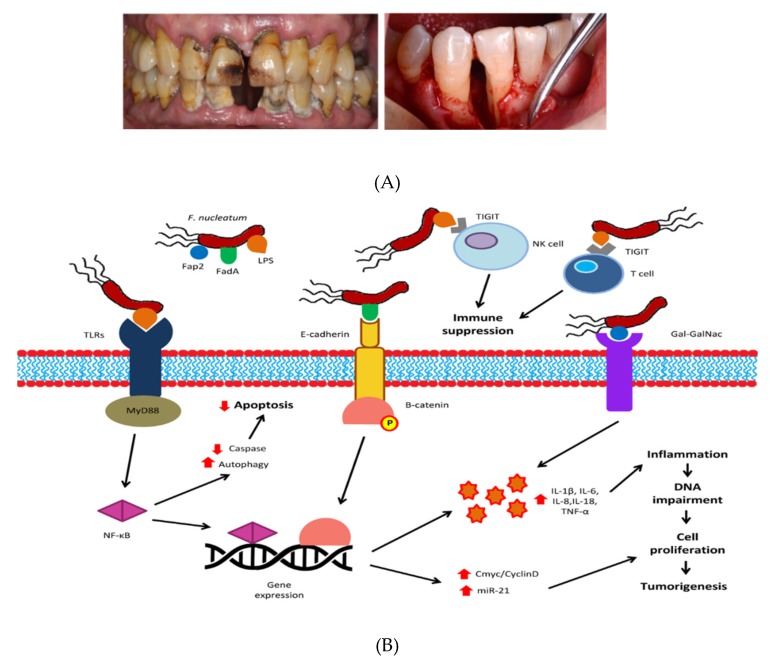
Periodontal disease patients clinically diagnosed (**A**) and schematic summary of the molecular pathways of *F. nucleatum* in CRC tumorigenesis (**B**). CRC: colorectal cancer; Gal-GalNAc: D-galactose-β (1–3)-N-acetyl-D-galactosamine; IL: interleukin, LPS: lipopolysaccharide; miR: microRNA: NF-κB: nuclear factor kappa-beta; NK: natural-killer; TIGIT: T-cell immunoglobulinand immunoreceptor tyrosine-based inhibitory motif domain; TLR: toll-like receptor; TNF-α: tumor necrosis factor-alpha. Upward red arrows: enhancement/stimulation; Downward red arrows: reduction (taken from Koliarakis et al., 2019 [37]).

At the oral level, several bacteria groups, such as *Fusobacterium nucleatum*, *Porphyromonas (P. gingivalis*), *Peptostreptococcus*, *Prevotella*, *Parvimonas*, and *Gemella* genera, are also found to be associated with colon cancer. In addition, *Treponema denticola* and *Prevotella intermedia* seem to be associated with increased CRC risk. In this sense, a complete description of the oral bacteria related with the development of CRC has been recently reported by Koliarakis et al. (2019) [37]. Although many oral bacteria have been suggested for their involvement in one or most types of CRC, two well-known pro-inflammatory, invasive, anaerobic, oral pathogens, with evident associations with dental plaques and periodontitis as later colonizers, *Fusobacterium nucleatum* and *Porphyromonas* spp. (*P. asaccharolytica* and *P. gingivalis*) are the most consistently reported microorganisms able to promote the clinical and molecular features of CRC [38,39,40]. One of the earlier studies concerning the role of Fusobacteria in CRC demonstrated enriched sequences of *Fusobacterium* spp. (*F. nucleatum*, *F. mortiferum*, and *F. necrophorum*) in CRC tissues, while bacteria belonging to *Firmicutes* and *Bacteroidetes* phyla were decreased [41]. This reduction in intestinal microbial diversity, as well as the overabundance of *Fusobacterium,* has been reported as common microbiota characteristics of CRC patients [37]. Interestingly, longer overall survival time was observed in CRC patients with low levels of *F. nucleatum* than in subjects with either moderate or high levels of this species. Going one step further, other studies have shown a connection between the detection of *F. nucleatum* and a poorer prognosis in CRC cases [42], suggesting the involvement of this bacteria as a non-invasive biomarker for CRC screening [37]. On the other hand, it is remarkable the high similarity found between *F. nucleatum* strains in tumor tissue and saliva from patients with CRC, which highlights an oral origin and strengthens the hypothesis about an orally driven intestinal dysbiosis in CRC [43,44]. According to this, four mechanisms of action of oral microbiota in the pathogenesis of CRC have been proposed [37]: (i) Dissemination of the oral bacteria into the intestinal environment through continuous swallowing of oral bacteria or spreading via bloodstream and systemic circulation (bacteremia). In the second case, inflammatory conditions of the oral cavity (periodontitis) may facilitate bacteremia, since during periodontitis the periodontal vasculature is more dilated and proliferated as a result of chronic inflammation (Figure 1); (ii) the role of oral polymicrobial biofilms in CRC through the disruption of the colonic mucus layer which negatively affects colon immunity; (iii) the metabolic properties of oral bacteria in the colon, which are characterized by saccharolytic and proteolytic metabolism, are able to degrade the mucins and extracellular matrix in the colon, resulting in infiltration of the mucus layer and invasion into the mucosa through disruption of epithelial junctions [27]. In turn, the ongoing destruction of the host proteins induces a chronic inflammatory state. On the other hand, it is remarkable that colonic biofilms of oral bacteria can further impair the colon by synthesizing carcinogenic metabolites, ROS, and polyamines; and (iv) the presence of virulence factors are able to inhibit apoptosis and modulate inflammation and the immune response. In the specific case of periodontal *F. nucleatum* (Figure 1), three major components, the Faps2, FadA, and lipopolysaccharide (LPS) mediates its oncogenic properties. LPS can interact with Toll-like receptors (TLRs) (TLR2 or TLR4), activating the MyD88 and NF-κB pathway. This interaction leads to reduced caspase activity and increased autophagy, resulting in reduced apoptosis. Furthermore, FadA binds to E-cadherin, causing dephosphorylation and activation of β-catenin. NF-κB and β-catenin alter gene expression, increasing the synthesis of pro-inflammatory cytokines (IL-1β, IL-6, IL-8, IL-18, TNF-α) and upregulating oncogenic pathways of Cmyc/CyclinD and miR-21. The pro-inflammatory state is further enhanced by the binding of Fap2 to Gal-GalNAc. Additionally, the interaction of LPS with the TIGIT receptor of NK and T cells leads to the suppression of anti-tumor immunity. Eventually, these events create inflammation, which impairs DNA, promotes cell proliferation, and results in CRC tumorigenesis. 

On the other hand, preliminary animal and human studies indicate that the colonic microbiota may be affected by oral bacteria, such as *P. gingivalis*, leading to dysbiosis. In particular, long-term oral ingestion of *P. gingivalis*, similarly to periodontitis, may influence intestinal dysbiosis. Apart from *P. gingivalis*, other periodontopathogens, including *A. actinomycetemcomitans*, can also disseminate to the colon [37]. Therefore, this oral–colon link may constitute another route for oral bacteria-mediated systemic inflammatory responses, that needs to be properly explored. 

Adequate oral hygiene habits are the main strategy used to prevent the onset of these disorders (e.g., tooth brushing, flossing, mouthwashes rinsing). Dietary patterns, and in particular, dietary polyphenols, seem to modulate the composition of the oral microbiota suggesting plausible benefits in the prevention of periodontal diseases [27].

As far as the intestinal level is concerned, recently Tarashi et al. (2019) [45] revised most of the studies on gut bacteria detected in stool and tissue samples of CRC. Evidence has shown that the gut microbiome differs between healthy individuals and cancer patients, and that microbial diversity in cancer is reduced [46]. Among the intestinal bacteria associated with CRC, studies highlight members of Bacteroidetes (*Bacteroides fragilis* subgroup ETBS, *Odoribacter* spp., *Alistipes finegoldii*), Firmicutes (*Streptococcus gallolyticus*, *Peptostreptococcus anaerobious*, *Enterococcus faecalis* and *Streptococcus bovis*), and Proteobacteria (*Escherichia coli*, *Campylobacter jejuni* and *Helicobacter hepaticus*) phyla. Some of the mechanisms proposed to contribute on CRC development have been the production of a toxin (fragilysin) able to cleaves E-cadherin on colonocytes and increases mucosal permeability, the ROS production, and the interaction with DNAse activity [47]. In this context, the analysis of fecal microbiome composition in patients with cancer has shown a marked enrichment of *Ruminococcus*, *Oscillobacter*, and *Roseburia* [48]. Similarly, a study carried out by Flemer et al. (2017) [49] showed the abundance of this bacterial group in mucosa samples from patients with CRC. More recently, in a metagenomic study involving Chinese and Danish patients with CRC, two new species associated with CRC, *Parviromonas micra,* and *Solobacterium moorei* were identified [50]. In line with this, studies using mice with colon tumor-bearing and germ-free received stool transplants from human CRC patients have demonstrated the crucial role of the intestinal microbiota in the CRC development and adenoma [51,52]. 

Recently published works highlight the importance of multidimensional data in the description of deep disease phenotypes, including microbiome and immune profiling. The new perspectives of patient evaluation can use both basic laboratory and clinical data (i.e., discrete variables), and omics’ data (i.e., continuous variables). Recent studies have shown that metabolomics may produce important diagnostic information for the main intestinal diseases, to examine metabolic cooperation host/microbiota with respect to phenotype, pathology, and diet [53]. As examples of these strategies, in CRC patients, a GC–MS- approach allowed the identification of 17 metabolites associated with ammonia recycling, Asp and Trp metabolism, and protein biosynthesis, which are involved in tumorigenesis (i.e., Lys, heptanedioic acid, norvaline), while 42 metabolites, involved in Asp and Ala metabolism and protein biosynthesis, were not detectable in the CRC group. Targeted metagenomics identified 76 bacteria, including *Enterobacteriaceae*, *Proteobacteria*, and Fusobacteria as key discriminants of CRC, whereas *Clostridiales*, *Clostridia*, *Firmicutes*, *Lachnospiraceae*, *Faecalibacterium,* and *Ruminococcaceae* were only ascribed to healthy patients considered as controls (CTRLs). An integrated analysis correlated CRC-associated microbes with metabolites, such as polyamines (cadaverine and putrescine), producing more functional insights than single datasets [54].

## 3. Dietary Polyphenols, Their Interactions with Microbiota, and Their Potential Therapeutic Role in Cancer

**Figure 2:**
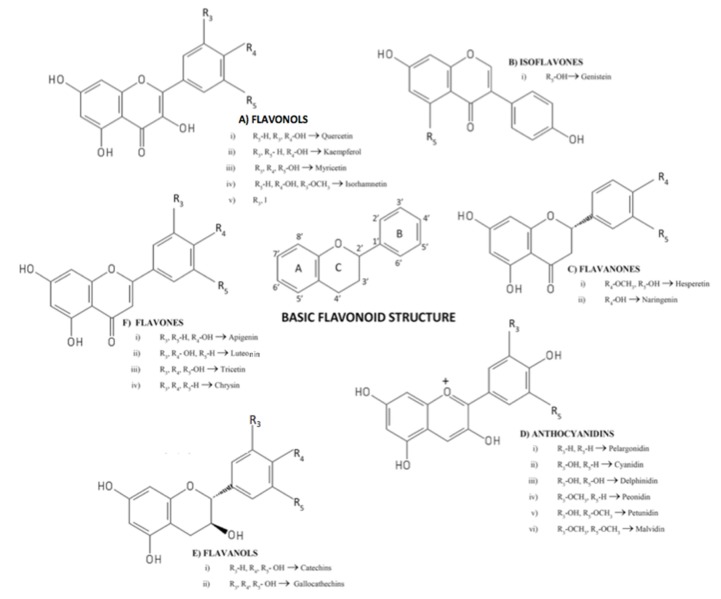
Chemical structures of the more common flavonoids in foods (reprints with permission of Santhakumar et al. 2018 [55]).

Polyphenols are a large group of compounds with high chemical diversity that are synthesized by plants for a variety of functions, such as protection against UV radiation, mechanical damage, and microbial infection. Chemically, phenolic compounds are characterized to have at least one aromatic ring with one or more hydroxyl groups attached. An exact classification and differentiation of these compounds is challenging due to their complex structures; >8000 identified compounds have been described to date, and classification is mainly based on their respective chemical composition. Figure 2 shows the chemical structures of the most common flavonoids in foods.

Polyphenols present in fruits, vegetables, and cereals have emerged as one of the main families of natural compounds; most of them have been considered functional foods with potential biological activities in many pathologies, such as cardiovascular diseases, diabetes, obesity and inflammation-related diseases, neurodegenerative disorders, and cancer [26,56]. Several health activities have been associated to dietary polyphenols, including antioxidant properties [57], anti-proliferative action [58], anti-inflammatory effects [59], anti-hypertensive and antithrombotic activities [60]. The main groups of dietary polyphenols promoting beneficial effects on human health are phenolic acids, flavonoids, stilbenes, and lignans [24,61].

**Figure 3:**
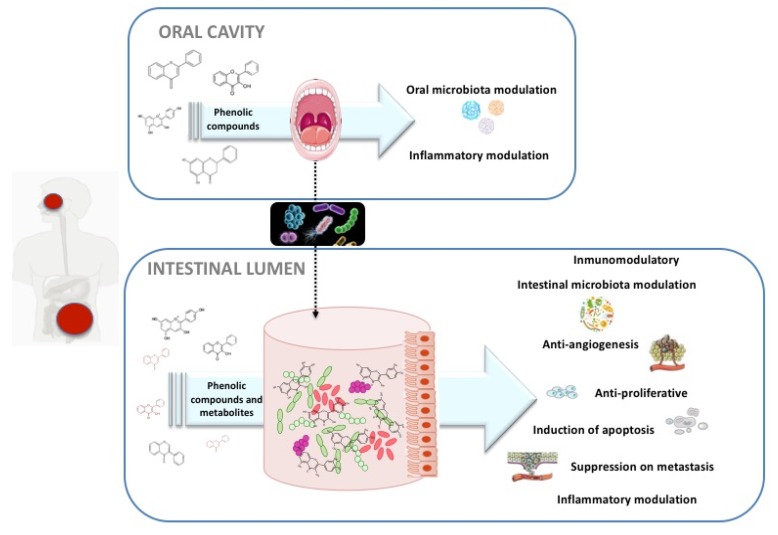
Schematic view about mechanism of action of polyphenols and their microbial metabolites and their therapeutic potential in CRC.

While the role of dietary polyphenols has been widely studied in the case of certain disorders, such as cardiovascular and metabolic diseases, the protective effect of these compounds in CRC is still in its infancy and little is known about the mechanisms of action involved. The role of dietary polyphenols in human health depends largely on their metabolism, absorption, and bioavailability, processes that are in turn intimately related to the modulation of the microbiota, both at composition and functionality levels. In the case of the oral ecosystem, whilst evidence demonstrates that the antimicrobial activity of polyphenols against certain periodontal pathogens is increasing, other mechanisms of action, including anti-adherent ability [62,63], inhibition of virulent enzymatic systems, and anti-inflammatory action, need to be evaluated to understand whether this orally-driven disruption promotes aberrant immune and inflammatory responses, eventually leading to CRC tumorigenesis. Many polyphenols have little bioavailability and reach the colon almost unaltered. There, they encounter the gut microbes, resulting in a two-way interaction in which polyphenols modulate the gut microbiota composition, and the intestinal microbes catabolize the ingested polyphenols to release metabolites that are often more active and better absorbed than the native phenolic compounds [64,65]. Since the two-way interaction between polyphenols and gut microbiota has been well-established, it is becoming clearer that the individuals’ gut microbiota can influence the potential effects of polyphenols in human health. Taking these physiological effects into account, the dual role of dietary polyphenols at the oral and intestinal microbiota levels, with a focus on the metabolism of polyphenols as a central axis, is reviewed below, with special emphasis on the mechanisms of action that could be involved in the prevention of CRC (Figure 3).

### 3.1. Effects of Polyphenols in CRC through Modulation of Oral Microbes

Polyphenol metabolism starts in the oral cavity. Whilst the metabolic events are unknown, they are capable of modulating the host inflammatory response and have been used as a chemopreventive agent to modulate the carcinogenesis process [27,66]. A possible way of preventing CRC is linked to the antimicrobial power of polyphenols, which can inhibit the oral bacterial growth, including *F. nucleatum* and *P. gingivalis* [67], as well as the adhesion to oral cells and enzymatic activity (enzymes are considered as virulence factors). Additionally, a decreased secretion of pro-inflammatory and increased secretion of anti-inflammatory cytokines has been recently reported by Bunte et al. (2019) [63]. As a result, many of the research studies in this area have focused on the use of polyphenols as antiadhesion therapy, which is considered an efficient way to prevent or treat bacterial infections and bacteremia. Although the effect of oral bacteria metabolites on CRC is unknown, in the case of *F. nucleatum* adherence, some phenolic compounds with antimicrobial and immunomodulatory characteristics, such as *p*-coumaric acid present in grape and red wine extracts, exert a stronger inhibition of pathogen adherence to oral cells, as shown by various recent studies, including those of our group [62]. These antiadhesive activities may be enhanced by the combined use of grape phenolic compounds and oral probiotics, preventing the progression of periodontal disease [68]. Flavonoids, especially flavan-3-ols and proanthocyanidins from green tea and grape and wine extracts appear to be the most promising candidates to be used in prevention or management of periodontal diseases [63,69]. Although this does not mean that the consumption of flavonoid-enriched foods reduces the risk of progression or prevents CRC, the antibacterial and anti-inflammatory effects of dietary polyphenols with regard to the inhibition of periodontal pathogen activities and the reduction in the host inflammatory and immune responses warrant further studies aimed at identifying the putative activities in relation to their contribution to colorectal carcinogenesis and uses in nutritional interventions for cancer prevention. 

### 3.2. Direct and Gut-Microbiota-Modulating Mechanisms of Action of Polyphenols against CRC at Intestinal Level

Once ingested, it has been estimated that 90–95% of total polyphenols reach the colon. Therefore, only 5–10% of the total polyphenol intake could be absorbed in the small intestine [70], where the polyphenols are transformed by the resident microbiota into bioavailable metabolites that might be even more bioactive than their precursors [71,72]. Depending on the specific chemical structure of the polyphenol and differences/variations in gut microbiota, an individual variability in metabolite production has been reported [73,74]. Dietary polyphenols and/or their metabolites may exert beneficial health effects at a local level, at a directly level during their passage through the oral cavity and gastrointestinal tract, and at a systemic level after being absorbed. The modulation of intestinal microbiota, anti-inflammatory effects, and the modulation of immune function could be the most remarkable beneficial local health effects exerted by polyphenols and their microbial metabolites. At the systemic level, potential cardio- and neuro-protective effects of polyphenols have been described, as well as a beneficial role in the prevention of some types of cancer, metabolic disorders, inflammatory diseases, and diabetes [23,75]. In this context, most of the mechanisms of action of polyphenols and their metabolites, which can positively influence the development of CRC, have been associated with anti-inflammatory and immunomodulatory activities.

The anticancer potential and chemoprevention of polyphenols for cancer is a very innovative topic in the field of food, nutrition, and health, and have been recently reviewed by several authors [76,77,78]. Preliminary studies in different animal models, human epidemiological trials, and cell lines indicate a correlation between polyphenolic intake an prevention of a particular cancer, showing a decreased risk for different types of cancer or diminished recurrence of cancer after the consumption of flavonoids, especially in the case of CRC [77,78,79]. In Table 1, we observe most relevant studies on the action of polyphenols in connection to CRC. Most of the trials were carried out in vitro using cellular models such as HT-29 and RAW 264.7 cells; we also found some tests performed in vivo in rats and mice [80,81,82,83,84]. However, it is only very recently that the first study that indicated that the protective effects of dietary polyphenols may be due to their metabolites [85]. 

In the anti-CRC and chemopreventive potential of polyphenols, different modes of action have been reported, principally as immunomodulatory and anti-inflammatory, as already indicated. Focusing on the immunomodulatory activity, several works conclude that the antitumor effect of polyphenols is due to the modulation of adaptive immune cells’ functionality in recognizing and lysing tumor cells, enhancing the immune response of dietary polyphenols. Blueberries rich in anthocyanins [80], red grapes [81] and cocoa [82] rich in condensed tannins, pomegranate extracts rich in hydrolysable tannins, and many other polyphenols showed these antitumor activities [83,84,90,91,92,93]. This effect significantly suppressed the secretion of several cytokines expression (for example IL-1β, TNF-α, IL-6, IL-17, and IFN-γ) and reduced the colonic infiltration of CD3+T cells producing these cytokines and neutrophils, and contributed to preventing adenoma formation. On the other hand, several authors explain that polyphenols’ anticancer activity is due to their anti-inflammatory and antioxidant properties, mainly mediated by their ability to down-regulate the nuclear factor NF-*k*B, modulating crucial cell signaling pathways involved in inflammation and cancer, and subsequently reducing the expression of IL-8 signaling in lipopolysaccharide-stimulated CRC cells [85,86,87,88,90,92]. 

Synergistic effects between different compounds have been described. For example, curcumin has been found to synergize with the natural agent resveratrol, both of which are known the anti-CRC effect; the combination therapy of curcumin and resveratrol is highly effective in inhibiting the growth of CRC cells in vitro and in vivo [94]. Another example is the combination of quercetin and piperine, a flavonoid and non-flavonoid compound; when combined, they represent an effective and potent anti-inflammatory effect by suppression in the secretion of TNF-α and interfere with the onset of IBD [26].

An appropriate diet can maintain the equilibrium between the inflammatory pathway and the anti-inflammatory process induced by many dietary compounds including polyphenols. Daily ingestion of polyphenols with fruits, vegetables, cereals, extra virgin olive oil, wine, tea, and coffee has also prompted further studies on their anti-cancer activity. Green tea, curcumin, polyphenols [epigallo-catechin-gallate (EPGC)], resveratrol, and quercetin are the most effective anti-cancer compounds as they inhibit NF-kB activation [95].

A recent population case-control study (*n* = 499) showed that a higher intake of total polyphenols, total flavonoids, total phenolic acids, anthocyanin, and flavanols was related to the decreased risk of CRC [96]. The biological effects for different native polyphenols have been discussed in several systematic papers (Table 1). Most of these anti-inflammatory, anticancer, and antioxidant activities have also been extrapolated to in vivo conditions using rodent models. However, it should be highlighted that in addition to the biological effects of the phenolic compounds in the digestive tract, they also exert health benefits indirectly through modulation of the microbiota, as mentioned above. This modulation includes both the effect of polyphenols on microorganisms considered as pathogens as well as their beneficial effect on the commensal microbiota (prebiotic effect). According with this, several in vitro and in vivo studies have demonstrated the role of polyphenols in inhibiting the growth of *Clostridium* spp. *(C. histolyticum), Pseudomonas* spp., *Salmonella* spp., *Bacillus* spp., *E. coli*, *H. pylori,* as well as increasing some beneficial bacterial groups, such as *Lactobacillus* spp., *Bifidobacterium* spp., *Akkermansia* spp. (*A. muciniphila*) and *Faecalobacterium* spp. (*F. prausnitzii*), some of which are able to metabolize polyphenols [97,98,99]. Based on this, we hypothesize that the inhibitory effect could be extensible to some of the previous intestinal bacteria proposed as associated with CRC; however, for most of them, further evidence is necessary to confirm these results and evaluate how these modifications could influence cancer progression. As an example, studies with food polyphenols have demonstrated their ability to inhibit the growth of *H.pylori* by different mechanisms that include, among others, the inhibition of urease activity and the interference with the key toxin of this bacteria (VacA) and DNA and RNA biosynthesis [98]. 

On the other hand, as mentioned previously, the modulation of microbiota may lead to changes in the metabolite profile expressed by gut microbiota that includes both the so-called potential oncometabolites; such as secondary bile acids, N-nitroso compounds, trimethylamine, and hydrogen sulfide, which are related with the consumption of red and processed meat and animal products high in saturated fat and cholesterol in the diet; and the potential tumor-suppressive metabolites, such as short chain fatty acids (SCFA: i.e., butyrate, propionate, and acetate) and phenolic compounds [100], mainly coming from the consumption of food polyphenols and fiber-rich plant food in the diet. Interestingly, some of microorganisms involved in the production of compounds considered as potential oncometabolites, such as *F. nucleatum* [8], are inhibited by polyphenols, as previously mentioned. In contrast, other microorganisms considered important for maintaining the normal balance of gut microbiota and gut barrier function, such as *Faecalibacterium* spp., increase after the intake of polyphenol-rich foods (i.e., red wine, pomegranate) [101] by the action of microbial phenolic metabolites. In relation with this, it has been also reported that the prebiotic effect exerted by phenolic metabolites increases the production of SCFA which are able to suppress the growth of some pathogens, function as energy sources for colonocytes and other bacteria (cross-feeding), dampen inflammation, and promote apoptosis of cancer cells. In fact, the lower abundance of beneficial bacteria SCFAs producers have been associated with CRC [8]. Therefore, the manipulation of SCFA levels in the intestine, through changes in microbiota, could be a potential preventive/therapeutic strategy for CRC [102]. However, at present, only preclinical evidence is available, and the effects have not been achieved in human studies yet. This is not the case of ellagitannins, for which, despite the fact that preclinical studies suggested their good potential use against CRC, a recent clinical trial in CRC patients showed no clear correlation between the phenolic fraction and the modulation of CRC-related markers in both normal and tumor colonic tissues after consumption of ellagitannin-rich pomegranate extracts [103,104]. In humans, the evidence that supports the activity of polyphenol-derived microbial metabolites in CRC is, to the best of our knowledge, non-existent. Dietary polyphenols can reach concentrations in the range of high µm to low mM in the gastrointestinal tract, whereas their gut microbiota-derived metabolites can be found in colon at concentrations up to high µM, and in other human systems at concentrations from nM to low µM [23]. It should also be taken into account that the gut-microbial derived metabolites often exert higher activity than their precursors, as shown for the antiproliferative activity of urolithins, the microbial derived metabolites from pomegranate [105]. Moreover, very recently, the stratification of individuals according to their ability to produce specific microbial-derived metabolites has been established for wine polyphenols [65,106], yet the further impact on health has been hardly approached. To date, the highest, albeit still limited, evidence that correlates a given gut metabotype with specific polyphenol health effects, using randomized clinical trials, has only been reported for isoflavones [107] and ellagitannins [108]. Interestingly, in relation to ellagitannins, metabotype B has been reported to be more prevalent in overweight/obese individuals as well as in metabolic syndrome and colorectal cancer patients [24].

Taken together, these findings suggest that polyphenols and their derived microbial-metabolites could be used as an interesting and complementary therapy against the complex development of CRC. Since the type and quantity of the polyphenols metabolites produced in humans depend on the gut microbiota composition and functionality, and different metabotypes have been identified, stratification in clinical trials according to individuals’ metabotypes is necessary to fully understand the health effects of dietary polyphenols.

## 4. Conclusions and Future Directions

The current review reports the most relevant recent epidemiological and experimental studies about the role of oral and intestinal microbiota in the pathogenesis of CRC. In turn, the effect of dietary polyphenols as chemopreventive agents in CRC, both directly and through the modulation of the composition and activity of oral and intestinal microbiota, is also discussed. From the microbiota’s perspective, a higher abundance of oral periodontal *Fusobacterium nucleatum,* as well as an enrichment of intestinal *Ruminococcus*, *Oscilobacter*, *Roseburia*, *Parviromonas micra*, and *Solobacterium moorei,* has been suggested as biomarkers of CRC [48,49,50]. On the other hand, the higher plasma homovallinic acid concentrations [109] and the higher activity of β-glucuronidase [79], a microbial enzyme from intestinal bacteria related with the polyphenols metabolism, have been recently associated with greater risk of CRC. These findings highlight that, in the future, the oral and intestinal microbiota could be used as novel markers of clinical significance to differentiate healthy and tumor patients, while natural compounds, such as polyphenols, can be used for preventive care. 

Nowadays it is considered that phenolic compounds contribute, at least in part, to the protective effects of fruit and vegetable-rich diets against cancer, hence, the study of their role in human nutrition has become a central issue in food research. In the future, more studies are needed to deeply evaluate the immunomodulatory role of dietary polyphenols and their metabolites in improving the efficacy of preventive and therapeutic capabilities, which it is likely to be an axis of increased interest in the coming years in relation to CRC and, in general, in the design of nutritional interventions for cancer prevention. In addition, it is expected that the knowledge about the role of the gut microbiota on polyphenol metabolism and its further impact on CRC will advance rapidly. In this sense, identifying specific biomarkers for the disease prediction in a global population may not be straightforward. It is notably important to consider the large inter-individual variability in polyphenol metabolism, and therefore stratification studies are needed in clinical trials to unambiguously identify the main factor responsible for the effects observed after polyphenols interventions. 

As it has been shown that oral and gut microbiota can be really important for the diet–cancer association, it will become essential to clarify the microbiota-mediated anti-cancer molecular mechanisms and to verify the promising therapeutic potentials of targeting microbiota by dietary polyphenols. In particular, it will be important to better understand the role of gut microbiota in the complex signaling cascades regulating host immune responses connected to CRC. Additionally, considering that the phenolic metabolites produced in humans depend on the gut microbiota composition and function, and different responses to polyphenols consumption depending on the individual have been identified, a current and future matter of research will be to identify the potential interplaying factors surrounding the interrelationships of polyphenols–gut microbiota and CRC and, as such, can pose a specific target for the development of potential nutritional and pharmaceutical approaches. Data from microbiomics integrated with patient metadata and other omics, such as metabolomics, metaproteomics, immunomics (proteomics and integrated serology), and lipidomics, may provide advanced clinical decisions for each CRC disease phenotype as well as the ability to design computer applications based on artificial intelligence to manage omics data and dietary profiles with the purpose of clinical-nutraceutical interventions.

## Figures and Tables

**Table 1:** Some studies reporting the effects of dietary polyphenols in CRC.

Polyphenol Source	Study	Mechanism	Conclusion	Reference
Blueberries	In vivo	Anti-inflammatory activity decreases mucosal levels of the proinflammatory cytokines, NF-a, IL- 1b, and IL-4 Reduced expression of inflammatory markers	Potential use of pterostilbene for CRC prevention	[80]
Red grapes	In vivo	Anti-inflammatory effects Suppressed the secretion of several cytokines (IFN-γ and TNF-α) Reduced tumor incidence and multiplicity	Resveratrol is a useful, nontoxic, complementary and alternative strategy to abate colitis and potentially CRC associated with colitis	[81]
Cocoa	In vivo	Anti-inflammatory activity decreases the nuclear levels of NF-κB and the expression of pro-inflammatory enzymes such as cyclo-oxygenase-2 Down-regulate the levels of inflammatory markers	Cocoa polyphenols suppress inflammation-related colon carcinogenesis and could be promising in the dietary prevention of CRC	[82]
Turmeric	In vitro	Biochemical changes to mesenchymal-epithelial transition Anti-proliferative activities	Curcumin might be a potential therapy for CRC and suppress metastasis	[86]
Cocoa	In vivo	Decreased the tumor incidence and size Suppressed colon tumorigenesis Induces apoptosis by increased the expressions of Bax and caspase 3 and decreased Bcl-xl.	Cocoa polyphenols may be a potential agent in the prevention and treatment of CRC specially in colitis type	[83]
Blueberries	In vitro	Significantly inhibited the growth of HCT116 and HT29 human CRC cells Anti-proliferative activities by inhibition apoptosis in CRC cells	Pinostilbene may play important roles in the anti CRC effects	[87]
Olive oil	In vitro	Anti-inflammatory effects Anti-proliferative activities by inhibition apoptosis in CRC cells	Cancer chemopreventive properties for CRC cells.	[88]
Japanese quince (*Chaenomeles japonica*)	In vitro	Anti-inflammatory effect by reduction COX-2 mRNA and MMP-9 protein expressions Inhibition of its enzymatic activity	Japanese quince polyphenols have cytotoxic, anti-inflammatory, and anti-metastatic activities towards the CRC cells	[89]
Cocoplum (*Chrysobalanus icaco L.*)	In vitro	Anti-inflammatory effect by decreased TNF-α, IL-1β, IL-6, and NF-κB1 Anti-proliferative activities in decreased the cell proliferation	Cocoplum anthocyanins possess cancer-cytotoxic and anti-inflammatory activities in CRC cells	[90]
Extra virgin olive oil	In vitro	Anti-inflammatory effects in inhibiting H_2_O_2_ production, GSH decrease, IL-6, and IL-8 Modulation of p38 and JNK MAPK/NF-κB signaling axis Oxysterols effects	Protective effect at intestinal level of extra virgin olive oil polyphenols, able to prevent CRC	[91]
Cranberries (*Vaccinium macrocarpon*)	In vivo	Suppresses colon tumorigenesis Anti-inflammatory effects in decreased of pro-inflammatory cytokines IL-1β, IL-6, and TNF-α Modulates multiple signaling pathways/proteins related to inflammation, cell proliferation, apoptosis, angiogenesis, and metastasis in the colon	Chemopreventive effects on colon tumorigenesis in mice	[84]
Sugarcane	In vitro	Anti-inflammatory effects by suppress the phosphorylation of NFκB and inhibit secretion of the pro-inflammatory cytokine IL-8. Regulation of important signaling proteins such as PKA, PKCβ, c-Jun, EGFR, and SIRT1	Chemopreventive effects on CRC	[92]
Mushrooms (*Pleurotus eryngii*)	In vitro	Anti-inflammatory effect by inhibiting the overproduction of pro-inflammatory cytokines Suppressed cell proliferation Induced cell cycle arrest and led to extensive cellular apoptosis in human CRC cells	*Pleurotus eryngii* polyphenols as a promising preventive agent against inflammatory disease and CRC	[93]
Cranberries (*Vaccinium macrocarpon*)	In vitro	Anti-inflammatory effects in inhibiting LPS-induced production of nitric oxide in macrophages Inhibition of the production of nitric oxide in macrophages Anti-cancer capacities in HCT116 cells and stronger inhibitory effects on the viability and colony formation capacity of HCT116	Non extractable polyphenol fraction showed promising anti-inflammation and anti CRC potential	[85]
